# Assessment of the endothelial function with changed volume of brachial artery by menstrual cycle

**DOI:** 10.1186/s12938-016-0230-x

**Published:** 2016-09-06

**Authors:** Shing-Hong Liu, Jia-Jung Wang, Da-Chuan Cheng, Chun-Hung Su, Tzu-Hsin Lin

**Affiliations:** 1 Department of Computer Science and Information Engineering, Chaoyang University of Technology, Taichung, Taiwan; 2 Department of Biomedical Engineering, I-Shou University, Kaohsiung, Taiwan; 3 Department of Biomedical Imaging and Radiological Science, China Medical University, 91, Xueshi Road, Hsueh-Shih Road, Taichung, 40402 Taiwan; 4 Institute of Medicine, School of Medicine, Chung-Shan Medical University, Taichung, Taiwan; 5 Department of Internal Medicine, Chung-Shan Medical University Hospital, No.110, Sec.1, Jianguo N. Rd., Taichung, 40201 Taiwan; 6 Department of Cardiology, Lin-Shin Hospital, Taichung, Taiwan

**Keywords:** Endothelial function, Flow-mediated vasodilation, Ultrasonography

## Abstract

**Background:**

The endothelial function has been proven to be an important factor in the pathogenesis of atherosclerosis, hypertension and heart failure. The flow-mediated vasodilation (FMD) of the peripheral artery is an endothelium-dependent function. Brachial-artery ultrasound scanning is the popular method for evaluating FMD. However, good technical training on ultrasonography is required for the user to obtain high-quality data. Therefore, the goal of this study was to propose a new method which only used a sphygmomanometer cuff to occlude the blood flow and record the vascular volume waveform (V_wave_).

**Results:**

We used this method to assess the FMD in the menstrual cycle for 26 volunteer females. All female subjects were evaluated two times (M: menstrual phase; F: luteal phase) in one menstrual cycle and for two cycles. In the first cycle, the FMD volume ratio in M was 101.9 ± 45.5 % and was higher in L, at 137.5 ± 62.1 % (p = 0.0032 versus M). In the second cycle, the FMD volume ratios in M and L were 91.4 ± 37.0 % and 124.0 ± 56.4 %, respectively (p < 0.001 vs. M).

**Conclusions:**

Our results have confirmed those results in the study of Hametner et al. Blood pressure measurement and FMD assessment all used the same mechanic of digital blood pressure monitor, which makes our method suitable using at home.

## Background

Endothelial dysfunction is considered an important factor to estimate the risk of atherosclerosis, hypertension, and heart failure [1–3]. Several measurement techniques have been used to assess the endothelium-dependent vasodilation, which includes the coronary epicardial vasoreactivity, coronary microvascular function, flow-mediated vasodilation (FMD), and peripheral arterial tonometry (PAT) [3–5]. Some studies have been proposed for intima-media thickness measurement [6, 7]. Currently, the most widely used noninvasive technique to assess endothelial dysfunction is the FMD ratio of the brachial artery, which was first introduced in 1992 [8]. The diameter changes of the brachial artery are induced by the hyperemia stimulus as a result of the mediated local endothelial releasing the nitric oxide (NO) [9, 10]. In order to create the hyperemia stimulus, a sphygmomanometer cuff is placed on the forearm. Then, the cuff inflates to the supra-systolic pressure to occlude the blood flow of the brachial artery in a standardized time that causes consequent ischemia dilation of the downstream obstructed vessels. Afterward, the cuff deflates and induces a brief high-flow state that causes the dilation of brachial artery.

The most used method to assess the FMD ratio is brachial-artery ultrasound scanning (BAUS), which requires a two-dimensional imaging process algorithm, an synchronous electrocardiogram monitor and a broadband linear array transducer (multiple-frequency: 7–14 MHz) [9, 10]. However, this method has some problems that must be overcome. The diameter of the brachial artery is measured from the continuous two-dimensional gray-scale imaging, which must have the clear anterior and posterior intimal interface between the lumen and the vessel wall. Thus, the linear array transducer must have good resolution [9]. Moreover, an individual training in the principles and technical skills of ultrasonography would affect the performance of the FMD measurement because the maximal diameter occurs approximately at 45–60 s after the hyperemic stimulus. The operator has to do the training in the technique who could get the high quality and consistency in the FMD measurement [11, 12]. Thus, Corretti et al. applied a training protocol for the ultrasound method [9].

In recent years, the measurement of the peripheral vasodilator response with fingertip PAT technology (EndoPAT, Israel) has been used to assess the endothelial dysfunction [8–10]. Although the fingertip PAT signal is interfered by various local, systemic, and environmental factors, this parameter is also affected by the bioavailability of NO; therefore, it also depends on the endothelial function [13–15]. The advantage of this method is its ease of use for the subjects, but it also requires a sphygmomanometer cuff placed at the forearm to create a hyperemic stimulus. In order to obtain a maximum volume change by the photoplethysmography, the finger-mounted probes include a system of inflatable neoprene membranes within a rigid external case to apply a counter-pressure (approximately 70 mmHg) for the finger. Thus, the change of finger arterial volume could be easier measured.

Moreover, Kuvin et al. compared the accuracy between PAT and BAUS for subjects with high risks of endothelium dysfunction. The results showed a significant correlation between the two parameters [15]. Some studies have proven that the postmenopausal women supplying the ovarian hormones could decrease the occurrence of atherosclerosis [16, 17]. Thus, Hashimoto et al. used the BAUS to assess the FMD of 17 volunteer women in three different phases of one menstrual cycle. They had shown that ovarian hormones could have a positive effect to reduce the endothelial dysfunction in humans. [18].

The changes of peripheral arterial volume are used to assess the endothelial function whether with BAUS or PAT methods. However, how to easily detect the change of arterial volume is still a challenge. Liu et al. proposed a method of constructing the “cuff model” that could detect the change of arterial volume by a sphygmomanometer cuff [19]. Moreover, they also compared the correlation of the changed volume measured by the cuff model and BAUS. The results showed that two methods have a high consistency [20]. Therefore, the goal of this study is to measure the FMD with this method which only uses one sphygmomanometer cuff to create the hyperemic stimulus and measure the FMD ratio. We also proposed a method to extract stable volume amplitudes and the maximum volume amplitude from the continuous waveform before and after the hyperemia stimulus. Twenty-six female volunteers participated in this study for two complete menstrual cycles. In one menstrual cycle, we measured the participants’ FMD ratios in the menstrual phase and luteal phase and compared the difference.

## Methods

The mechanism of our measurement system was based on a digital blood pressure monitor. The measurement system first measured the blood pressure and subsequently measured the FMD ratio. We will describe the experimental protocol in “Experimental protocol” section.

### Hardware

Our system had a pressure sensor and a flow sensor. Both the pressure sensor (MPM 100, range: 0–300 mmHg, resolution: 16 bits, Metrodyne Microsystem Corp., Taiwan) and the flow sensor (MFS 100, range: 0.3 to 2 l/minute, resolution: 12 bits, Metrodyne Microsystem Corp., Taiwan) were digital [20]. A TI MSP 430 F6736IPZ was used to perform the peripheral interface control and detect the cuff pressure and cuff volume [20]. The sampling rate was 125 Hz. The systolic and diastolic pressures could be detected using our designed instrument in one measurement. Moreover, the measured signals from the sensors during the inflating and deflating cycles were transmitted to a notebook computer by bluetooth.

### Volume of the brachial artery

In the oscillometric measurement, Liu et al. used a pressure sensor and a flow sensor to construct the cuff model (*C*_*cuff*_) during the inflating cycle [19]. According to the cuff model, the pulse pressure (*P*_*wave*_), extracted from the pulse signal of the cuff pressure could be transferred to the pulse volume (*V*_*wave*_). Equation (1) shows the transfer under a cuff pressure (*P*_*cuff*_),

1 $$ V_{wave} \left( {P_{cuff} } \right) = C_{cuff} \left( {P_{cuff} } \right) \cdot P_{wave} \left( {P_{cuff} } \right) . $$

### FMD ratio

**Fig. 1:**
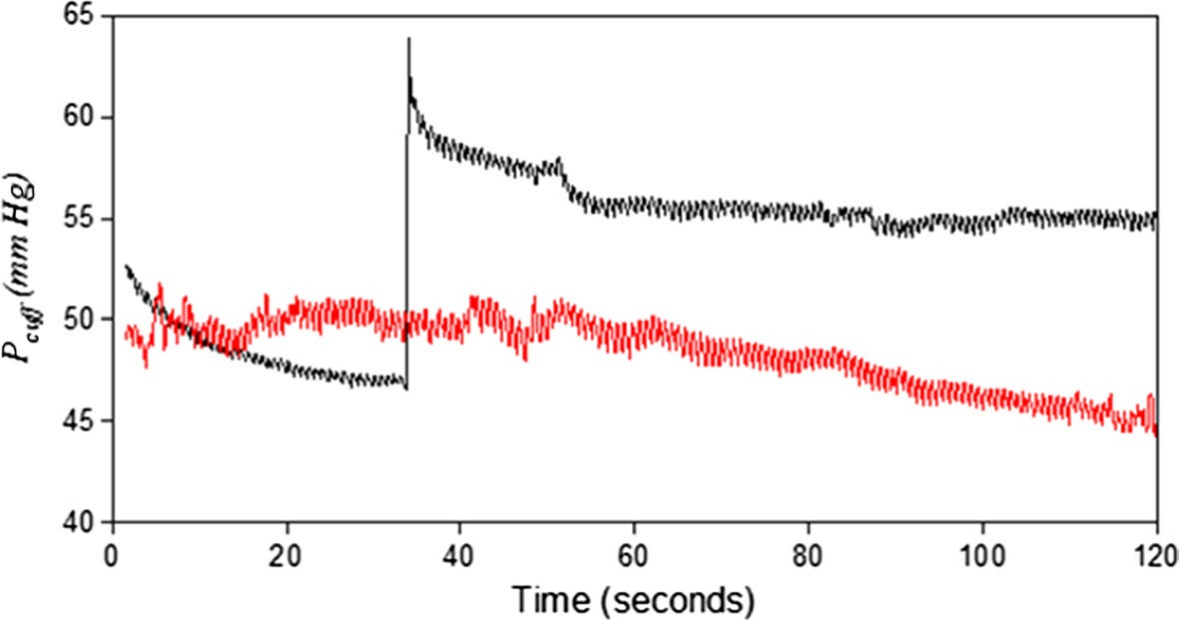
The *P*_*cuff*_ signals before (*black*) and after (*red*) the hyperemic reaction

**Fig. 2:**
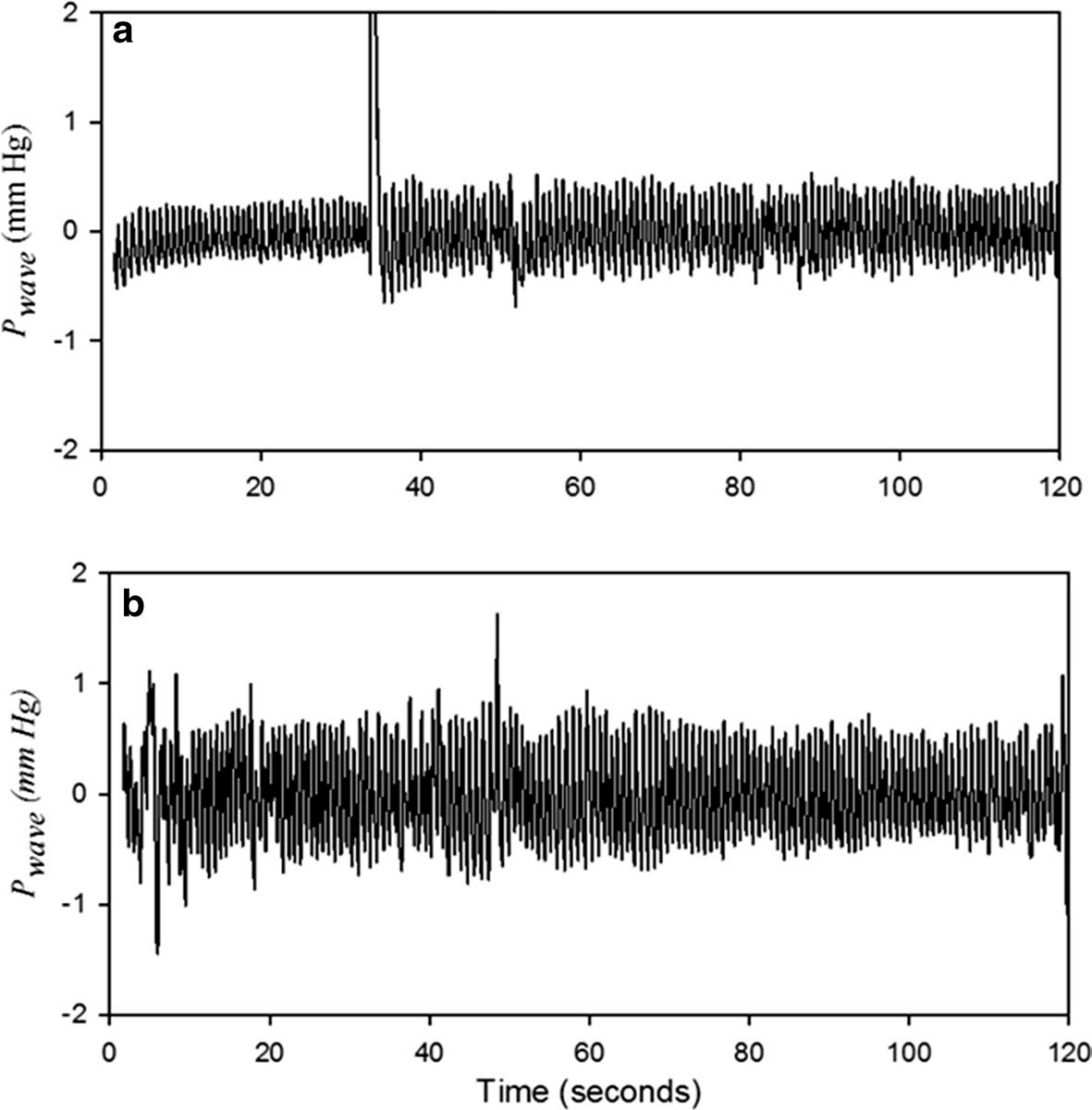
The filtered *P*_*wave*_ signals from Fig. 1, **a** before, **b** after hyperemic reaction

**Fig. 3:**
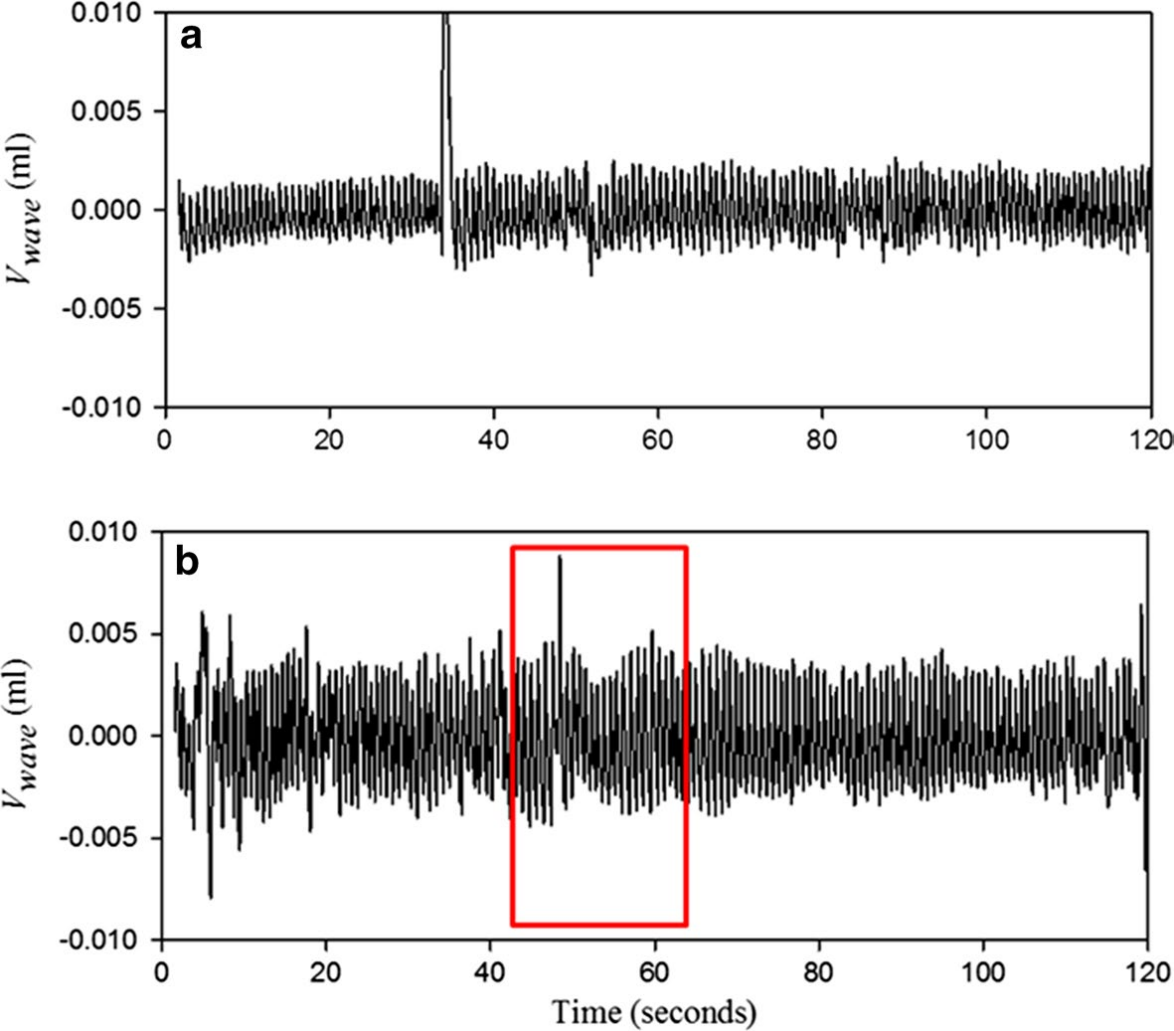
The *V*_*wave*_ signals transferred from Fig. 2

In Fig. 1, the red line shows *P*_*cuff*_ in the duration of the baseline and the black line shows *P*_*cuff*_ in the duration of the hyperemia reaction for one subject. Moreover, only the amplitude of *V*_*wave*_ (Δ*V*) needs to be detected in the measurement of FMD ratio. Thus, the *P*_*wave*_ signals were filtered from the *P*_*cuff*_ signals in the durations of the baseline and hyperemia reaction as shown in Fig. 2a, b, respectively. The order of the high-pass filter was one, and its cutoff frequency was 0.5 Hz. The order of the low-pass filter was two, and its cutoff frequency was 40 Hz. The filtered pressure waveforms Then, according to Eq. (1), *P*_*wave*_ was transferred to *V*_*wave*_. Figure 3 shows the *V*_*wave*_ signals in the duration of the baseline and hyperemia reaction.

The *P*_*cuff*_ was remained stable at a fixed pressure, the first phase was to search the maximum amplitude of the pulse volume in the duration of the hyperemia reaction (ΔV_*hyperemia*_) and the cuff pressure of ΔV_*hyperemia*_ was marked. The second phase was to search for stable amplitudes of pulse volume around the marked cuff pressure in the duration of the baseline. The average of five consecutive stable amplitudes of pulse volume was defined as the baseline (Δ*V*_*baseline*_) for the FMD measurement.

**Fig. 4:**
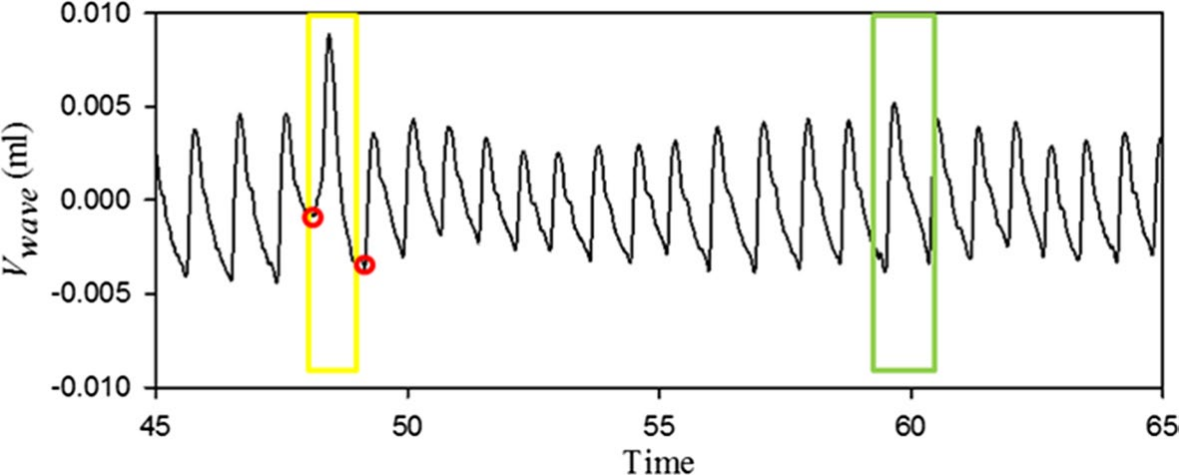
The detailed *V*_*wave*_ signal from the *red range* of Fig. 3b, a false *V*_*wave*_ is marked by a *yellow rectangular mark*, a truth *V*_*wave*_ is marked by a *green rectangular mark*

To avoid extracting a false *V*_*wave*_, we defined a true *V*_*wave*_ as having an evident exponential decay in a diastolic duration [21, 22] and a limited difference between the systolic starting point and the diastolic ending point. Figure 4 shows the signal at 45–65 s of Fig. 3b in more detail. Although there is maximum amplitude at the 48th second, which is marked with a yellow rectangle, the *V*_*wave*_ value of this beat was affected by the body motion because its difference between the systolic starting point and the diastolic ending point (marked with red circles) is notably large. The truth maximum amplitude occurs at the 60th second, which is marked with a green rectangle.

The normalized difference of two neighboring truth amplitudes of pulse volume is defined as follows,

2 $$ \Delta V_{diff} = \frac{{2\left[ {\Delta V\left( n \right)\, - \Delta V\left( {n - 1} \right)} \right]}}{\Delta V\left( n \right) + \Delta V(n - 1)} \times 100\,\% , $$

where *n* is the nth beat. Then, we defined the stable Δ*V*(*n*) as Δ*V*_*diff*_ within ±10 %. The volume FMD ratio is defined as follows,

3 $$ FMD_{volume} = \frac{{\left| {\Delta V_{hyperemia} \,{ - }\, \Delta V_{baseline} } \right|}}{{\Delta V_{baseline} }} \times 100\,\% . $$

### Experimental protocol

#### Subjects

The FMD ratio was assessed using our method for 26 voluntary women with ages of 26.3 ± 10.0 years (mean ± SD; range: 19–45 years), all of whom were healthy students and staff members of the school, and did not eat any oral contraceptives. We follow the protocol in [18] to select our female subjects. These female subjects had regular menstrual cycles (26–35 days) for more than 3 months before this study [18]. They were not pregnant and were normotensive, non-diabetic, and nonsmoking during this study. The subject provided written informed consent before participating in this study after thorough explanation of the study design and protocol.

#### Study design

Each female subject has been measured two times for two cycles in one menstrual cycle. The menstrual phase M and luteal phase L were examined per cycle. In the menstrual phase, the FMD was measured on the second day of menstruation. The luteal phase was defined as 5–7 days after an obvious increase of morning body temperature. To estimate their menstrual cycles, the participants checked their body temperature every morning. According to the method described by Corretti et al. the subjects did not eat or drink caffeine, high-fat foods, intake vitamin C or use tobacco for at least 4–6 h before the study [9]. Thus, we asked the subjects to measure the FMD before breakfast. If a subject ate breakfast, we canceled the measurement and asked her to return for measurement the next day. If a participant failed twice, the measurement of her menstrual cycle was not possible. Therefore, only 23 subjects finished the FMD measurement for both menstrual cycles.

#### Measurement of FMD ratio

**Fig. 5:**
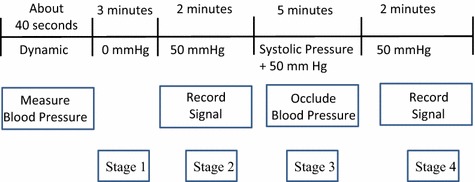
The time sequence of the FMD measurement, the* first row* is the spending time, the* second row* is the *P*_*cuff*_, and the* third row* is the functions of this system in the different stages

**Fig. 6:**
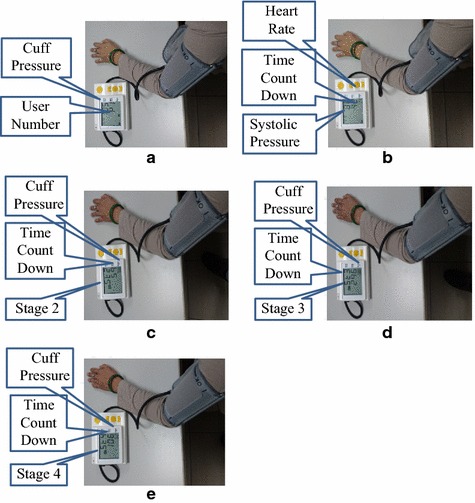
The real photo for FMD measurement, **a** blood pressure measurement, **b** stage 1, waiting 3 min, **c** stage 2, recording signal in 2 min, **d** stage 3, occluding blood flow for 5 min, **e** stage 4, recording signal in 2 min

The study was performed according to the method described by Corretti et al. in a quiet and temperature-controlled (22–23 °C) room [9]. The examinations were conducted by the same examiner throughout this study. The subjects sat on a chair, and their left arm was placed on the table, as with the blood pressure measurement. Because our designed device could directly detect blood pressure, the FMD-measuring procedure was automated as shown in Fig. 5. The first row is the duration of each stage. The second row describes the amount of pressure in the cuff. The third row describes the functions for the various stages. Subjects were required to rest for approximately 3 min before the measurement. First, the blood pressure of the subject was measured whose systolic pressure was used to set the cuff pressure for occluding the blood flow. Then, the subject rested for 3 min in the first stage. In the second stage, the cuff was inflated to about 50 mmHg, and the pulse volume signal was recorded for 2 min. The blood flow was occluded for 5 min in the third stage. The cuff was inflated to at least 50 mmHg above systolic pressure. In the fourth stage, the cuff was deflated to about 50 mmHg, and the pulse volume signal was recorded for 2 min. Figure 6 shows the real photos for FMD measurement, and Fig. 6a shows the procedure of blood pressure measurement. Figure 6b shows the procedure of the first stage, the time counts down from 180 to 0 s. Figure 6c shows the procedure of the second stage. The time counts down from 120 to 0 s, and the cuff pressure is also displayed. Figure 6d, e show the procedures of third and fourth stages.

## Results

**Fig. 7:**
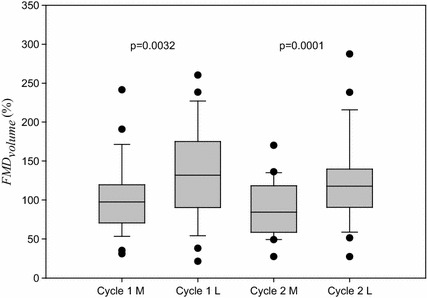
The *bar graphs* show the comparisons between the two phases in the two cycles

The baseline data of the subjects’ variables are presented in Table 1. We used the paired *t* test to compare the information of each subject. The *FMD*_*volume*_ ratios in the luteal phase (137.5 ± 62.1 and 124.0 ± 56.4 %) were higher than those in the menstrual phase (101.9 ± 45.5 and 91.4 ± 37.0 %), and there were significant differences between the menstrual and luteal phases (p = 0.0032 and p = 0.000). Figure 6a shows the bar graph of *FMD*_*volume*_. However, the blood pressure of the subjects did not show any difference between the two phases. We considered the effect of the *P*_*cuff*_ difference between the baseline and hyperemia reaction stages on the assessment of FMD ratio. Figure 7 shows the correlation between the *P*_*cuff*_ difference and *FMD*_*volume*_ in the first menstrual cycle. *P*_*cuff*_ difference and *FMD*_*volume*_ did not have any correlation (M: p = 0.938, L: p = 0.836).

**Table 1:** Baseline hemodynamic data of the important variables to characterize the subjects

	First cycle (n = 26)			Second cycle (n = 23)		
	M phase	L phase	p	M phase	L phase	p
Age (years)	26.3 ± 10.0					
BMI (kg/m^2^)	22.3 ± 1.8					
Sys. (mmHg)	106.8 ± 8.9	105.5 ± 10.1	0.318	105.2 ± 8.6	106.9 ± 9.8	0.218
Dia. (mmHg)	68.2 ± 7.0	67.9 ± 7.9	0.725	67.6 ± 5.3	68.7 ± 7.2	0.444
Temp. (°C)	36.1 ± 0.51	36.4 ± 0.48	0.000***	36.2 ± 0.31	36.5 ± 0.24	0.000***
*FMD*_*volume*_ (%)	101.9 ± 45.5^a^	137.5 ± 62.1^b^	0.0032**	91.4 ± 37.0^a^	124.0 ± 56.4^b^	0.000***

*Sys.* systolic pressure, *Dia.* diastolic pressure, *Temp.* temperature

* p < 0.05, ** p < 0.005, *** p < 0.0001

^a^First cycle (M phase) vs second cycle (M phase), p = 0.374

^b^First cycle (L phase) vs second cycle (L phase), p = 0.432

## Discussions

In previous studies, the FMD ratio of the peripheral artery was usually assessed using the BAUS and PAT techniques [8–10, 13–15]. Moreover, the FMD induced by reactive hyperemia is known to depend on the presence of endothelial cells [4, 5, 23]. Therefore, a sphygmomanometer cuff was used to occlude the blood flow for the assessment of FMD ratio. To inflate the cuff to a supra-systolic pressure, these techniques must first measure the blood pressure of the subjects. Therefore, we combined the two functions, assessment of FMD ratio and blood pressure measurements, in one instrument. The sphygmomanometer cuff not only was used to occlude the blood flow, but also to detect the amplitude of *V*_*wave*_ by the cuff model [19]. In order to evaluate the performance of our method, we follow the experiment protocol of Hashimoto et al. to measure the FMD ratios of 26 female subjects in the menstrual and luteal phases. The results also showed that the *FMD*_*volume*_ ratio in the luteal phase was the significant increase than the menstrual phase. The results were same as the study of [18]. Moreover, we designed the repeated measurement.

Some studies used the pressure waveform to assess the FMD ratio. Wu et al. used the pulse pressure of radial artery to detect the FMD ratio for elderly diabetics [24, 25]. Using ensemble empirical mode decomposition, a significant signal for the response of FMD was extracted from the raw pulse pressure waveform. In their studies, Wu et al. used a sphygmomanometer cuff at the forearm to create the hyperemia stimulus. A wrist cuff, which was inflated at 40 mmHg, was used to record the pulse pressure waveform. But, according to the theorem of FMD measurement, the peripheral arterial volume or blood flow before and after the hyperemic stimulus would change. Although, the amplitude of arterial volume waveform is proportional relation to the amplitude of arterial pressure waveform according to the arterial model [26, 27], the relation is a nonlinear function. When the transmural pressure is too high or low, the change of arterial pressure waveform cannot represent the change of arterial volume waveform [28]. Therefore, the hypertension or hypotension subjects would not suggest using the pulse pressure waveform to assess the FMD ratio.

**Fig. 8:**
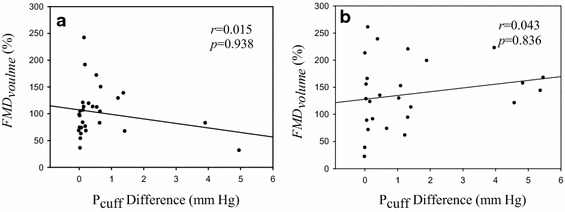
The correlation between *P*_*cuff*_ difference and *FMD*_*volume*_, **a** is in the menstrual phase, **b** is in the luteal phase

The disadvantage of using the cuff to detect the FMD ratio of the brachial artery is that it is difficult to remain *P*_*cuff*_ at a constant value because *P*_*cuff*_ is easily affected by motion artifacts. *P*_*cuff*_ in the baseline stage usually slowly decreases, possibly because the inner material of the cuff is polyvinyl chloride (PVC) or silicon, which is an elastic material. When the cuff is tightly wound around the forearm, the inner layer of the cuff contacting with the skin has many folds on. The folds would slowly disappear when the cuff is inflated to a low pressure. Thus, the cuff volume increases, and the cuff pressure decreases. To overcome this problem, the cuff is inflated again when *P*_*cuff*_ decreases to 46 mmHg, as shown in Fig. 1. In Fig. 1, *P*_*cuff*_ during the hyperemia reaction slowly increases and then decreases because the folds do not disappear when the cuff is quickly inflated to a higher pressure (above the systolic pressure). However, when *P*_*cuff*_ is deflated from the occluded stage to the hyperemia reaction stage, the cuff would slowly recover its elasticity and the folds disappear. But, in the hyperemia reaction stage, the cuff is not inflated again if *P*_*cuff*_ descends. Therefore, the *P*_*cuff*_ of the ΔV_*hyperemia*_ beat was set as the target *P*_*cuff*_ for searching stable *V*_*wave*_ beats in the baseline duration. The *P*_*cuff*_ of stable *V*_*wave*_ beats must be the closest to the target pressure. Although, we had proved that the *P*_*cuff*_ difference did not correlate with the FMD ratio in Fig. 8, we believed that the *P*_*cuff*_ difference between the baseline and hyperemia reaction stages could not be large.

In Table 1, The *FMD*_*volume*_ ratio in the second menstrual cycle varied between the two phases more than in the first menstrual cycle (first vs. second: p = 0.0032 vs. p = 0.000), which may be the reason there were only 23 subjects in the second menstrual cycle. Moreover, the comparison of menstrual phase or luteal phase between two cycles did not have the significant difference, which implied that the two menstrual experiments were independent. The temperature in the luteal phase was higher than in the menstrual phase regardless in the first cycle or the second cycle.

Atherosclerosis happening in the coronary artery and cerebral artery is very dangerous in the clinic. The endothelial dysfunction is a systemic condition [29]. According to Ross study, the vascular endothelial dysfunction is an initial step in the growth of atherosclerosis [30]. Thus, some studies have reported that patients with atherosclerosis in the coronary artery have the condition of the endothelial dysfunction in the brachial artery [31, 32]. Therefore, although the assessing method of FMD ratio is applied only to superficial arteries, like as brachial artery and the femoral artery, it could be used to assess the cardiovascular risk [5]. Now whether BAUS or EndoPat method could not be use by myself in the home. Thus, the advantage of using the cuff to detect the FMD ratio of brachial artery is that the digital blood pressure monitor could include the function of FMD ratio measurement and be operated by one person.

## Conclusions

The vascular endothelial dysfunction is an initial step in the growth of atherosclerosis. Reactive-hyperemia-induced FMD is known to depend on endothelium. The purpose of our study is to design an FMD measurement system with the same mechanic structure as a commercial digital blood pressure monitor. Therefore, the system can measure blood pressure and assesses vascular endothelial function based on the FMD ratio in the patient’s home.

## Abbreviations

FMD: flow-mediated vasodilation
PAT: peripheral arterial tonometry
NO: nitric oxide
BAUS: brachial-artery ultrasound scanning
*C*_*cuff*_: cuff model
*P*_*cuff*_: cuff pressure
*P*_*wave*_: pulse pressure
*V*_*wave*_: pulse volume
Δ*V*: amplitude of pulse volume
ΔV_*hyperemia*_: maximum amplitude of the pulse volume in the duration of the hyperemia reaction
Δ*V*_*baseline*_,: stable amplitudes of pulse volume in the duration of the baseline
Δ*V*_*diff*_: normalized difference of two neighboring truth amplitudes of pulse volume
FMD_volume_: FMD ratio

## References

[1] Schachinger V, Britten M, Zeiher A (2000). Prognostic impact of coronary vasodilator dysfunction on adverse long-term outcome of coronary heart disease. Circulation.

[2] Suwaidi J, Hamasaki S, Higano S (2000). Long-term follow-up of patients with mild coronary artery disease and endothelial dysfunction. Circulation.

[3] Deanfield JE, Halcox JP, Rabelink TJ (2007). Contemporary reviews in cardiovascular medicine. Circulation.

[4] Lekakis J, Abraham P (2011). Methods for evaluating endothelial function: a position statement from the European society of cardiology working group on peripheral circulation. Eur J Cardiovasc Prev Rehabil.

[5] Flammer AJ, Anderson T (2012). The assessment of endothelial function from research into clinical practice. Circulation.

[6] Cheng D, Lin JT (2012). Three-dimensional expansion of a dynamic programming method for boundary detection and its application to sequential magnetic resonance imaging (MRI). Sensors.

[7] Cheng D, Jiang X (2008). Detections of arterial wall in sonographic artery images using dual dynamic programming. IEEE Trans Inf Technol Biomed.

[8] Celermajer DS, Sorensen KE, Gooch VM, Spiegelhalter DJ, Miller OI, Sullivan ID (1992). Non-invasive detection of endothelial dysfunction in children and adults at risk of atherosclerosis. Lancet.

[9] Corretti MC, Anderson TJ (2002). Guidelines for the ultrasound assessment of endothelial-dependent flow-mediated vasodilation of the brachial artery: a report of the international brachial artery reactivity task force. J Am Coll Cardiol.

[10] Ghiadoni L, Versari D, Giannarelli C, Faita F, Taddei S (2008). Non-invasive diagnostic tools for investigating endothelial dysfunction. Curr Pharm Des.

[11] Stadler RW, Karl WC, Lees RS (1996). New methods for arterial diameter measurement from B-mode images. Ultrasound Med Biol.

[12] Stadler RW, Taylor JA, Lees RS (1997). Comparison of B-mode, M-mode and echo-tracking methods for measurement of the arterial distension waveform. Ultrasound Med Biol.

[13] Rubinshte R, Kuvin JT, Soffler M, Lennon RJ, Lavi S, Nelson RE, Pumper GM, Lerman LO, Lerman A (2010). Assessment of endothelial function by non-invasive peripheral arterial tonometry predicts late cardiovascular adverse events. Eur Heart J.

[14] Hamburg NM, Benjamin EJ (2009). Assessment of endothelial function using digital pulse amplitude tonometry. Trends Cardiovasc Med.

[15] Kuvin JT, Patel AR (2003). Assessment of peripheral vascular endothelial function with finger arterial pulse wave amplitude. Am Heart J.

[16] Manolio TA, Furberg CD, Shemanski L, Psaty BM, O’Leary DH, Tracy RP, Bush TL (1993). Associations of postmenopausal estrogen use with cardiovascular disease and its risk factors in older women. Circulation.

[17] Lieberman EH, Gerhard MD, Uehata A, Walsh BW, Selweyn AP, Ganz P, Yeung AC, Creager MA (1994). Estrogen improves endothelium-dependent, flowmediated vasodilation in postmenopausal women. Ann Intern Med.

[18] Hashimoto M, Akishita M, Eto M, Ishikawa M, Kozaki K, Toba K, Sagara Y, Taketani Y, Orimo H, Ouchi Y (1995). Modulation of endothelium-dependent flow-mediated dilatation of the brachial artery by sex and menstrual cycle. Circulation.

[19] Liu SH, Wang JJ, Huang KS (2008). A new oscillometry-based method for estimating the dynamic brachial artery compliance under loaded conditions. IEEE Trans Biomed Eng.

[20] Liu SH, Lin TH, Cheng DC, Wang JJ (2015). Assessment of stroke volume from brachial blood pressure using arterial characteristics. IEEE Trans Biomed Eng.

[21] Navakatikyan MA, Barrett CJ, Head GA, Ricketts JH, Malpas C (2002). A real-time algorithm for the quantification of blood pressure waveform. IEEE Trans Biomed Eng.

[22] Hametnera B, Wassertheurera S, Kropfa J, Mayer C, Holzingerc A, Eberd B, Weberd T (2013). Wave reflection quantification based on pressure waveforms alone—methods, comparison, and clinical covariates. Comput Methods Programs Biomed.

[23] Barac A, Campia U, Panza JA (2007). Methods for evaluating endothelial function in humans. Hypertension.

[24] Wu HT, Lee CH, Liu AB, Chung WS, Tang CJ, Sun CK, Yip HK (2011). Arterial stiffness using radial arterial waveforms measured at the wrist as an indicator of diabetic control in the elderly. IEEE Trans Biomed Eng.

[25] Wu HT, Lee CH, Sun CK, Hsu JT, Huang RM, Tang CJ (2012). Arterial waveforms measured at the wrist as indicators of diabetic endothelial dysfunction in the elderly. IEEE Trans Instrum Meas.

[26] Raamat R, Talts J, Jagomagi K, Lansimies E (1999). Mathematical modeling of non-invasive oscillometric finger mean blood pressure measurement by maximum oscillation criterion. Med Biol Eng Comput.

[27] Tyan CC, Liu SH, Chen JY, Chen JJ, Liang WM (2008). A Novel Noninvasive Measurement Technique for Analyzing the Pressure Pulse Waveform of the Radial Artery. IEEE Trans Biomed Eng.

[28] Liu SH, Tyan CC (2010). Quantitative analysis of sensor for pressure waveform measurement. Biomed Eng.

[29] Anderson TJ, Gerhard MD, Meredith IT, Charbonneau F, Delagrange D, Creager MA, Selwyn AP, Ganz P (1995). Systemic nature of endothelial dysfunction in atherosclerosis. Am J Cardiol.

[30] Ross R (1993). The pathogenesis of atherosclerosis: a perspective for the 1990s. Nature.

[31] Egashira K, Inoue T, Hirooka Y, Yamada A, Urabe Y, Takeshita A (1993). Evidence of impaired endothelium-dependent coronary vasodilatation in patients with angina pectoris and normal coronary angiograms. N Engl J Med.

[32] Uehata A, Gerhard MD, Meredith IT, Lieberman EH, Selwyn AP, Creager M, Polak J, Ganz P, Yeung AC, Anderson TJ (1993). Close relationship of endothelial dysfunction in coronary and brachial artery. Circulation.

